# Radiation forces of beams generated by Gaussian mirror resonator on a Rayleigh dielectric sphere

**DOI:** 10.1038/s41598-017-12406-3

**Published:** 2017-09-22

**Authors:** Bin Tang, Kai Chen, Lirong Bian, Xin Zhou, Li Huang, Yi Jin

**Affiliations:** 1 School of Mathematics & Physics, Changzhou University, Changzhou, 213164 China; 2 School of Sciences, Hunan University of Technology, Zhuzhou, 412008 China

## Abstract

Optical trapping and manipulating of micron-sized particles have attracted enormous interests due to the potential applications in biotechnology and nanoscience. In this work, we investigate numerically and theoretically the radiation forces acting on a Rayleigh dielectric particle produced by beams generated by Gaussian mirror resonator (GMR) in the Rayleigh scattering regime. The results show that the focused beams generated by GMR can be used to trap and manipulate the particles with both high and low index of refractive near the focus point. The influences of optical parameters of the beams generated by GMR on the radiation forces are analyzed in detail. Furthermore, the conditions for trapping stability are also discussed in this paper.

## Introduction

Optical trapping and manipulating of micron-sized particles have attracted enormous interests in optical tweezers because of the advantages of being noncontact and noninvasive since the pioneering work by Ashkin and his coworkers who first successfully captured a dielectric sphere by using a single laser beam^1^. This optical trapping has been applied in various areas including physics, chemistry, and biophysics^2^, and it can be used to manipulate kinds of tiny objects such as uncharged atoms and molecules^3^, living biological cells^4^, DNA molecules^5^, metallic spheres^6,7^, magnetodielectric particles^8^, and so on. As a consequence, optical tweezers have been developed into one of the most promising tools in micromanipulation from trapping to rotating and sorting^9–13^. It is well known that the mechanical action of light on particles is the consequence of exchange of momentum and energy between photons and particles^14,15^. However, the conventional optical traps or tweezers are constructed mainly by fundamental Gaussian beams. The ordinary Gaussian beams are limited not only in the kinds of particles, but also in the number of particles for capturing at one time. Meanwhile, the initial optical tweezer’s model is not suitable for trapping biological cells, because the light intensity at the center of the beam is too high and is very likely to kill cells. It has been confirmed that the radiation forces induced by a focused laser beam are closely related to the optical characteristics such as beam profile and polarization. In recent decades, researchers have demonstrated that trapping of particles with different refractive indices can be implemented by using different types of laser beams. For example, the particles with refractive index larger than the ambient medium can be trapped by flat-topped beams^16^, Lorentz-Gauss beam^17^, or Gaussian Schell-model beams^18^. By contrast, the particles with refractive index smaller than the ambient medium can be trapped by dark hollow beams^19–21^. Also, it has been shown that one can trap two types of particles with different refractive index using bottle beams^22,23^, Hermite-cosine-Gaussian beams^24^ or Laguerre-Gaussian beams^25,26^. In addition, the trapping characteristics of other beams, such as the radially and azimuthally polarized beams^27–29^, Airy beams^30^ and pulsed Gaussian beams^31^ have been explored by using the Rayleigh scattering theory. Meanwhile, optical forces on small particles from partially coherent light have been studied in terms of a partial gradient of the space-variable diagonal elements of the coherence tensor^32^. Further, by combining vortex with evanescent field, the evanescent wave can be utilized to realize subwavelength trapping^33,34^. As a result, the evanescent optical vortex field is able to trap 200 nm polystyrene spherical particles^33^. Furthermore, the particles ranging in sizes from 9.5 to 275 nm in diameter could be trapped in three dimensions using low laser power by minimizing spherical aberrations at the focus point^35,36^.

On the other hand, optical resonators with variable reflectance mirrors have been proposed and successfully implemented in many gas and solid-state lasers in recent years^37^. Particularly, the optical resonator with a Gaussian mirror offers advantages over standard unstable resonator of good mode discrimination, smooth output beam profile, and large mode volume. Furthermore, the beams generated by Gaussian mirror resonator (GMR) can be decomposed into a linear combination of the lowest-order Gaussian modes (TEM_00_) with different parameters^38^. Up to now, the propagation properties of beams generated by GMR have been extensively investigated in different optical media and systems^39–41^. However, to the best of our knowledge, the radiation forces of beams generated by GMR on particles have not been reported elsewhere.

In this paper, we have derived the analytical formulas for the beams generated by GMR propagating through a paraxial *ABCD* optical system. Based on the Rayleigh scattering theory, the radiation forces of beams generated by GMR acting on a Rayleigh dielectric sphere particle in the Rayleigh scattering regime are investigated numerically and theoretically. The influences of optical parameters of the beams generated by GMR on the radiation forces are analyzed in detail. In addition, the conditions of the stable trapping are also discussed under the Rayleigh approximation. The results show that beams generated by GMR can be used to trap and manipulate simultaneously the particles with both high and low index of refractive nearby the focus point of the lens system. Our results will have promising applications in optical trapping.

## Field distribution of a focused beam generated by GMR

In cylindrical coordinate system, the optical field distribution of a beam generated by GMR at the input plane (*z* = 0) can be expressed as^38^

1 $$E(r,0)={E}_{0}\,\exp (-\frac{{r}^{2}}{{w}_{0}^{2}}+{\rm{i}}k\frac{{r}^{2}}{2{R}_{0}}){[1-K\exp (-2{\beta }^{2}\frac{{r}^{2}}{{{w}_{0}}^{2}})]}^{1/2},$$

where *E*_0_ represents the amplitude of the beam, *r* is the radial coordinate, *R*_0_ is the wave-front curvature of the incident beam, the parameter *K* is called the on-axis (or peak) reflectivity of this mirror and *k* = 2π/*λ* is the wave number, *β* is a parameter that is given by *w*_0_/*w*_*c*_, *w*_*c*_ is the mirror spot size at which the reflectance is reduced to 1/*e* ^2^ of its peak value, and *w*_0_ is the beam waist. By using the binomial expansion method, Eq. (1) can be re-expressed as

2 $$E(r,0)=\sum _{m=0}^{\infty }{E}_{m}\exp (\frac{{\rm{i}}k}{2{q}_{m}}{r}^{2}),$$

with *E*_*m*_ = $${\alpha }_{m}{E}_{0}$$, *α*_0 _= 1, $${\alpha }_{1}=-K/2$$, and

3 $$\,{\alpha }_{m}=\frac{(2m-3)(2m-5)\ldots (3)(1)}{m!}{(\frac{K}{2})}^{m},m\ge 2.$$

In equation (2), some parameters are introduced by

4 $$\frac{1}{{q}_{m}}=\frac{1}{{R}_{0}}+\frac{{\rm{i}}\lambda }{{\rm{\pi }}{({w}_{0})}_{m}^{2}},$$

5 $${({w}_{0})}_{m}=\frac{{w}_{0}}{{(2m\beta +1)}^{1/2}},m=0,1,2,\cdots .$$

The electric field of the beam passing through a paraxial optical *ABCD* system without aperture can be calculated by the Collins formula, which takes the form as follows^39^

6 $$E({r}_{1},\theta ,z)=\frac{{\rm{i}}k}{2{\rm{\pi }}B}{\int }_{-\infty }^{\infty }{\int }_{0}^{2{\rm{\pi }}}E(r,0)\,\exp \{-\frac{{\rm{i}}k}{2B}[A{r}_{1}^{2}-2{r}_{1}r\,\cos (\phi -\theta )+D{r}^{2}]\}r{\rm{d}}r{\rm{d}}\phi ,$$

where *r*_1_, *θ* and *r*, *φ* are the radial and azimuthal angle coordinates in the input and output planes, respectively. The abbreviation of *ABCD* is a 2-by-2 matrix associated with an optical element which can be used for describing the element’s effect on a laser beam, and *A*, *B*, *C*, *D* are the transfer matrix elements of the paraxial optical system. After tedious integral calculations, one can obtain

7 $$\,E({r}_{1},\theta ,z)=\sum _{m=0}^{\infty }\frac{{E}_{m}{q}_{m}}{B+A{q}_{m}}\exp \,[\frac{{\rm{i}}k}{2}(\frac{C{q}_{m}+D}{A{q}_{m}+B}){r}_{1}^{2}].$$

**Figure 1:**
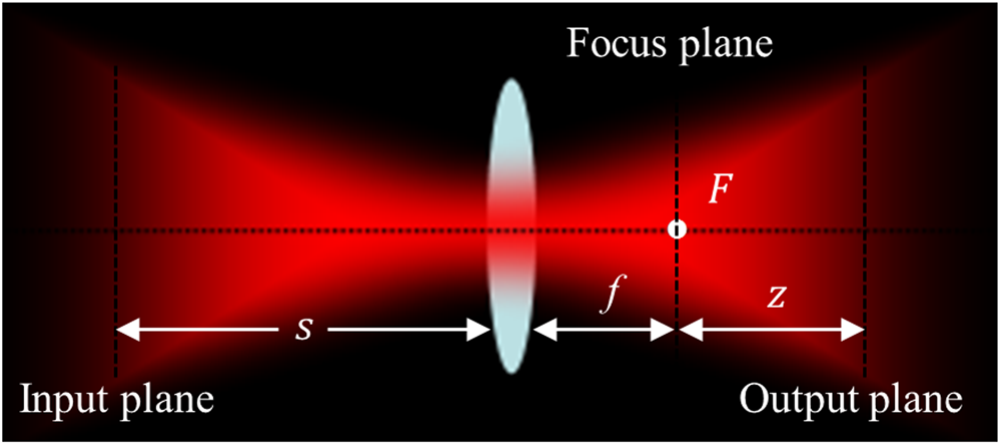
Schematic of an unapertured thin lens system.

Incidentally, we assume that beam waist *w*_0_ locates in the plane of the Gaussian mirror (assumed here to be at *z* = 0), namely *R*_0_ → ∞. Also, the following formulas are used in the derivation of Eq. (7)^42^

8 $${\int }_{0}^{2{\rm{\pi }}}\exp [-{\rm{i}}\xi \,\cos \,(\phi -\theta )]{\rm{d}}\phi =2{\rm{\pi }}{J}_{0}(\xi ),$$

9 $${\int }_{0}^{\infty }{x}^{\upsilon +1}\exp (-a{x}^{2}){J}_{\upsilon }(\mu x)dx=\frac{{\mu }^{\upsilon }}{{(2a)}^{\upsilon +1}}\exp (-\frac{{\mu }^{2}}{4a}),$$

where $\mathrm{Re}(a) > 0,\mathrm{Re}(\upsilon ) > -1$, and ${J}_{\upsilon }(\cdot )$ is the *υ*th-order of the first kind Bessel function. Now we consider the beams generated by GMR propagating through an unapertured lens as shown in Fig. 1, the transfer matrix for the lens system can be given by

10 $$(\begin{array}{cc}A & B\\ C & D\end{array})=(\begin{array}{cc}-z/f & (-z/f)s+f+z\\ -1/f & 1-s/f\end{array}),$$

where *f* is the focus length of the thin lens, *s* is the axial distance from the input plane to the thin lens, and *z* is the axial distance from the focus plane to the output plane. The point *F* in Fig. 1 is the focus point.

Substituting Eqs (7) into (10), we can obtain the field distribution of the beams generated by GMR through the thin lens in cylindrical coordinate system as follows:

11 $$\begin{array}{c}E({r}_{1},z)=\sum _{m=0}^{\infty }\frac{{E}_{m}}{-\frac{z}{f}+[(-\frac{z}{f})s+f+z]\,[\frac{{\rm{i}}\lambda }{{\rm{\pi }}{({w}_{0})}_{m}^{2}}]}\\ \,\,\,\,\,\,\,\,\,\,\,\,\,\,\,\,\,\,\,\,\times \,\exp \{\frac{i{\rm{\pi }}{r}_{1}^{2}}{\lambda }\frac{-\frac{1}{f}+(1-\frac{s}{f})\,[\frac{{\rm{i}}\lambda }{{\rm{\pi }}{({w}_{0})}_{m}^{2}}]}{-\frac{z}{f}+[(-\frac{z}{f})s+f+z]\,[\frac{{\rm{i}}\lambda }{{\rm{\pi }}{({w}_{0})}_{m}^{2}}]}\}.\end{array}$$

**Figure 2:**
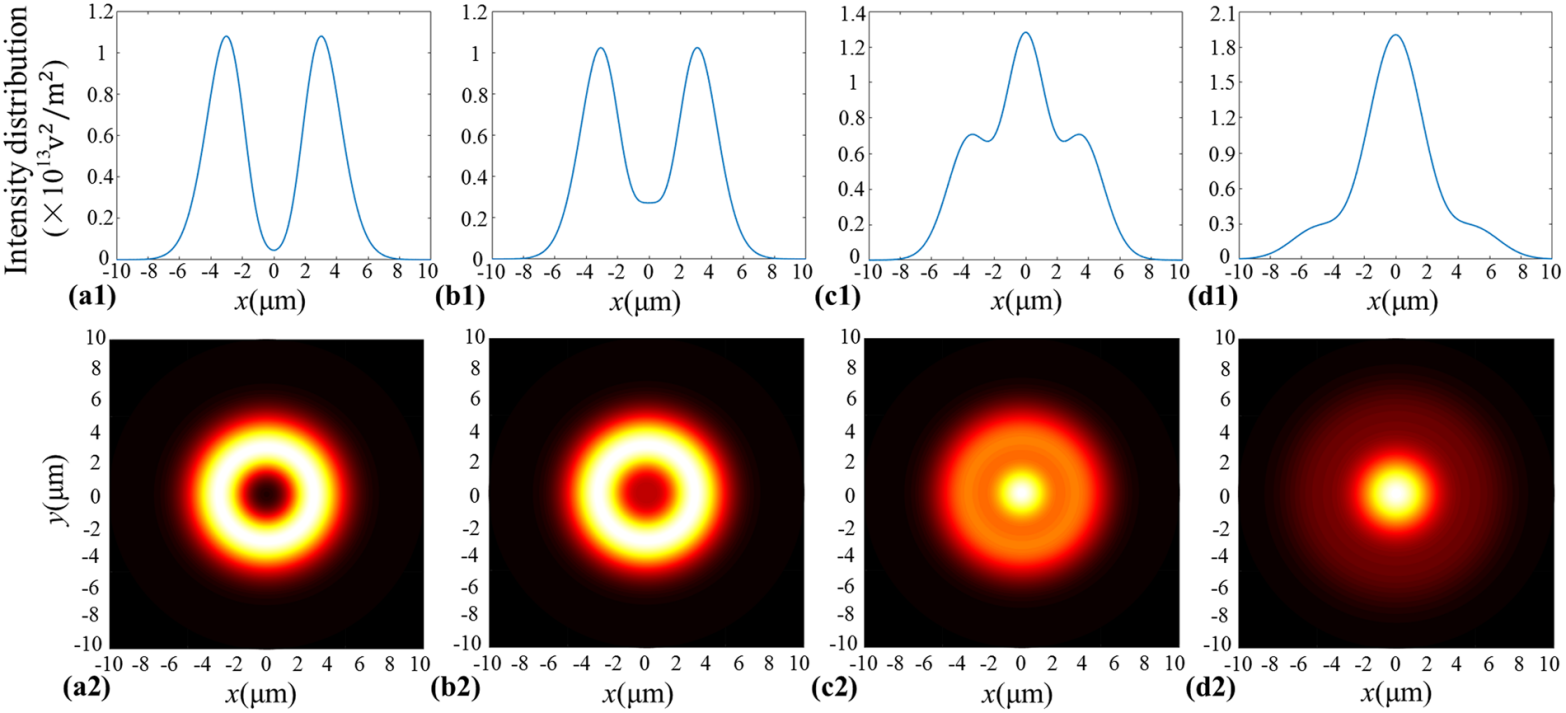
Intensity distributions of the beams generated by GMR at different positions. (**a1**) *z* = 0 µm, (**b1**) *z* = 5 µm, (**c1**) *z* = 15 µm, (**d1**) *z* = 30 µm. The bottom row (**a2**–**d2**) shows the corresponding contour of the intensity distribution.

In the following calculations, we choose *w*_0_ = 4 mm, *m* = 10, *λ* = 1.06 µm, *f* = 30 mm, *s* = 30 mm, *K* = 0.7, *β* = 0.8, and the input power of the beams generated by GMR is assumed to be 1 W, which always keep unchanged unless otherwise stated. In Fig. 2, we plot the intensity distributions of the beams generated by GMR at different propagation positions. From Fig. 2, one can find that the intensity of the output beam takes on a hollow Gaussian-like distribution which has two peaks at the focus plane (*z* = 0 µm). When the propagation distance is far away from the focus plane, the two peaks meet together and gradually become a sharp peak. Due to these special characteristic of the focused beams generated by GMR, one can expect that it is useful for trapping the microscopic particles by using the focused beams generated by GMR.

## Radiation forces of the focused beams generated by GMR

In this section, we discuss on the radiation forces produced by the focused beams generated by GMR on dielectric particles in Rayleigh scattering regime. For simplicity, we treat the particles as spheres and assume that the radius *a* of the particle is much smaller than the wavelength of incident light, i.e., *a* ≪ *λ*. For this case, the Rayleigh approximation is applicable. The radiation forces exerting on the particle in the Rayleigh regime include the scattering force and the gradient force. The scattering force ${F}_{{\rm{scat}}}$ is proportional to the intensity of incident light, which can be expressed as^15^

12 $${\mathop{F}\limits^{\rightharpoonup }}_{scat}(r,z)={\mathop{e}\limits^{\rightharpoonup }}_{z}{n}_{m}\alpha I(r;z)/c,$$

where ${\mathop{e}\limits^{\rightharpoonup }}_{z}$ denotes the unity vector along the direction of beam propagation, *n*_*m*_ is the refractive index of the ambient, $c=1/\sqrt{{\varepsilon }_{0}{\mu }_{0}}$ is the speed of the light in vacuum, *ε*_0_ and *μ*_0_ denote the dielectric constant and the magnetic permeability in the vacuum, respectively. $I(r,z)$ is the intensity of the focused beams and given by $I(r,z)={n}_{m}{\varepsilon }_{0}c{|E(r,z)|}^{2}/2$, and *α* is defined as the scattering coefficient as follows^15^

13 $$\alpha =\frac{128{{\rm{\pi }}}^{5}{a}^{6}}{3{\lambda }^{4}}{(\frac{{\chi }^{2}-1}{{\chi }^{2}+2})}^{2}.$$

where $\chi ={n}_{p}/{n}_{m}$, ${n}_{p}$ being the refractive index of the particle; For the gradient force *F*_grad_, it is induced by non-uniform optical field, and its direction is same as the gradient of light intensity. So, the gradient force *F*_grad_ is given by^15^

14 $${\mathop{F}\limits^{\rightharpoonup }}_{{\rm{g}}rad}(r,z)=\frac{2{\rm{\pi }}{n}_{m}{a}^{3}({\chi }^{2}-1)\nabla I(r;z)}{({\chi }^{2}+2)c}.$$

By using Eqs (12–14), we can calculate the radiation forces acting on a Rayleigh dielectric sphere produced by the focused beams generated by GMR. Without loss of generality, we select the radius of the particle *a* = 50 nm, the refractive index of the ambient *n*_*m*_ = 1.33 (e.g. water), and the refractive indices of two kinds of particles: *n*_*p*_ = 1.59 (e.g. glass particles in the water) or *n*_*p*_ = 1(e.g. the bubbles in the water) in the following calculations.

**Figure 3:**
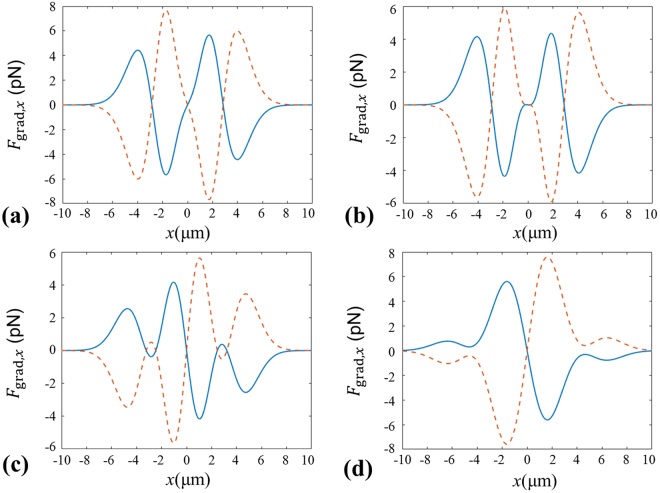
Transverse gradient force produced by the focused beams generated by GMR at different planes. (**a**) *z* = 0 µm, (**b**) *z* = 5 µm, (**c**) *z* = 15 µm, (**d**) *z* = 30 µm. Solid curves for the particles with *n*_p_ = 1.59, dashed curves for the particles with *n*_p_ = 1.

In Fig. 3, we depict the transverse gradient forces ${F}_{{\rm{grad}},x}$ at different propagation distances. Here, the positive value of the transverse gradient force means the direction of *F*_grad,x_ is along the positive direction of the *x*-axis; On the contrary, the negative value of the transverse gradient force signifies the direction of *F*_grad,x_ is along the negative direction of the *x*-axis. From Fig. 3(a) and (b), it is clearly seen that there is one stable equilibrium point near the focal point for the particles with *χ* < 1 as shown by the dashed line. Meanwhile, one can see that there exist two stable equilibrium points at about *x* = ±3 µm for the particles with *χ* > 1 as indicated by the solid curve. It means we can use the focused beams generated by GMR to trap or manipulate simultaneously the particles with both *χ* > 1 and *χ* < 1 nearby the focus point. With further increasing of the propagation distances, one can find from Fig. 3 (c) and (d) that there exists only one stable equilibrium point for the particles with *χ* > 1 due to the appearance of a sharp peak in the center of the ring as shown in Fig. 2. However, there exist no stable equilibrium points for the particles with *χ* < 1 when the position is far away from the focus point.

**Figure 4:**
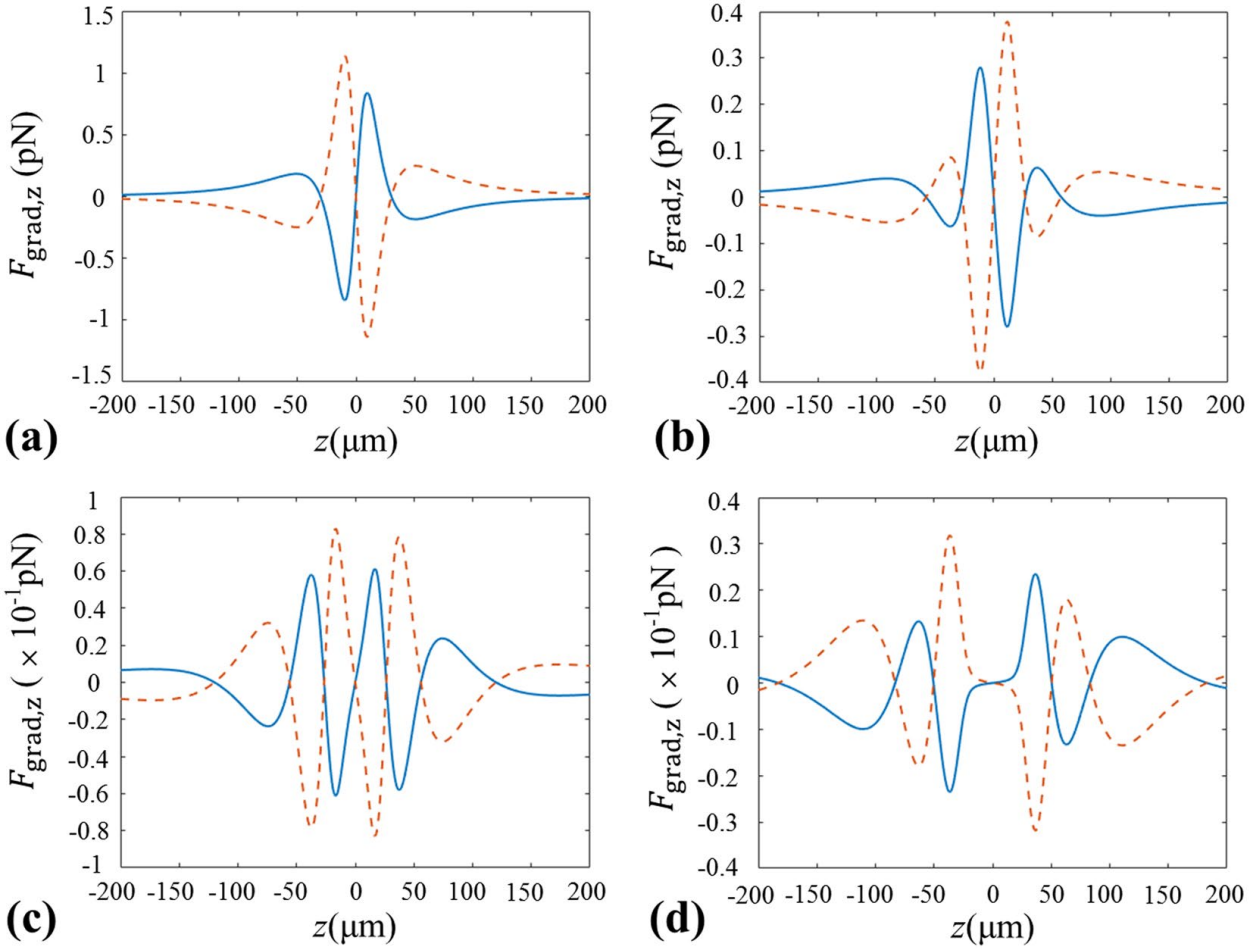
Longitudinal gradient force produced by the focused beams generated by GMR at different transverse positions. (**a**) *x* = 0 µm, (**b**) *x* = 3 µm, (**c**) *x* = 6 µm, (**d**) *x* = 9 µm. Solid curves stand for the particles with *n*_p_ = 1.59, and dashed curves are for the particles with *n*_p_ = 1.

In Fig. 4, we plot the changes of the longitudinal gradient force ${F}_{{\rm{grad}},z}$ at different transverse positions. Similarly, the longitudinal force is along +*z* (or −*z*) direction for the positive (or negative) ${F}_{{\rm{grad}},z}$. From Fig. 4(a), one can find that there is an equilibrium point for particles with *χ* < 1 at the focus point, which means we can trap or manipulate the particles with *χ* < 1 at the focus point. By contrast, the equilibrium point for particles with *χ* < 1 disappears and an equilibrium point for particles with *χ* > 1 comes up at *x* = 3 µm in Fig. 4(b). Furthermore, one can see From Fig. 4(c) and (d) that there appears one equilibrium point for particles with *χ* < 1 and two equilibrium points for particles with *χ* > 1 with increasing of the transverse distance, separately. And the longitudinal gradient force ${F}_{{\rm{grad}},z}$ decreases and the stability of equilibrium point becomes worse. The further discussions on the trapping stability will be given in the next part.

**Figure 5:**
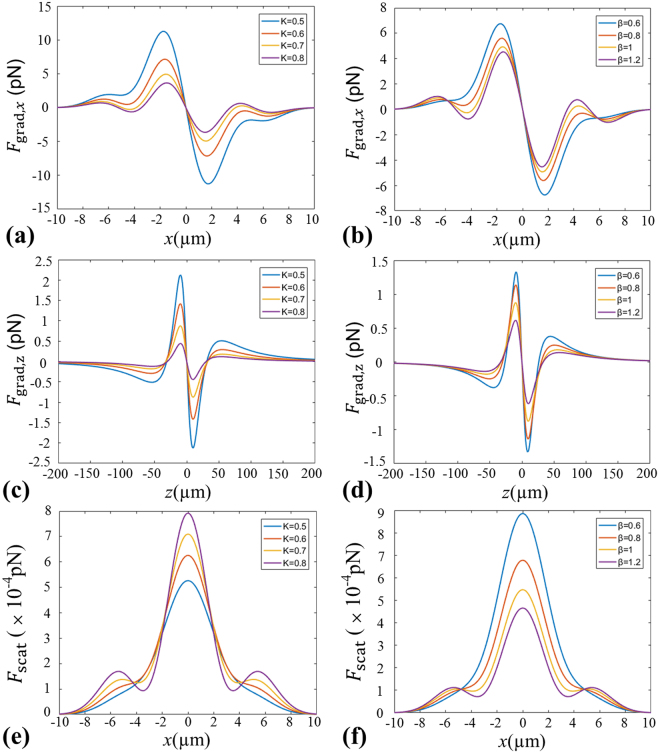
Radiation forces produced by the focused beams generated by GMR for different optical parameters. (**a**,**b**) The transverse gradient force acting on a particle with refractive index *n*_p_ = 1.59 at *z* = 30 µm. (**c**,**d**) The longitudinal gradient force acting on a particle with refractive index *n*_p_ = 1 at *x* = 0 µm. (**e**,**f**) The scatting force acting on a particle with lower refractive index *n*_p_ = 1 at *z* = 30 µm.

To further study the influences of optical parameters (e.g. *K* and *β*) of the beams generated by GMR on the radiation forces, Fig. 5 illustrates the evolutions of optical radiation forces for the beams generated by GMR with optical parameters *K* and *β* when taking different values. In this calculation, the parameter *β* is fixed by 0.8 when *K* is changed from 0.5 to 0.8. By contrast, the parameter *K* takes a fixed value 0.7 when *β* is changed from 0.6 to 1.2. From Fig. 5(a) and (b), it can be found that the transverse trapping range becomes smaller and the trapping stability reduces gradually with increasing the values of *K* and *β*, respectively. Here, the refractive index of the dielectric particle is still assumed to be *n*_p_ = 1.59, and the trapping range is the distance from the equilibrium position to the position where trapping starts to happen, and the trapping stability means the maximum value of the force in the trapping range. In addition, it should be reminded that there only exists one equilibrium point for particles with *χ* < 1 at the focus point as seen from Fig. 4(a). Therefore, we just give in Fig. 5(c) and Fig. 5(d) the longitudinal gradient force acting on a particle with lower refractive index, i.e. *n*_p_ = 1. One can see from the pictures that the longitudinal trapping range almost keeps unchanged but the trapping stability reduces as the value of *K* or *β* goes up. Figure 5(e) and (f) display the scatting force acting on a particle with refractive index *n*_p_ = 1 for different parameters *K* and *β*, respectively. One can see that the parameters *K* and *β* have a distinctive influence on the scattering force. The scattering force increases with increasing of the value *K*. On the contrary, the scattering force decreases as the value of *β* increases.

## Discussion on trapping stability

**Figure 6:**
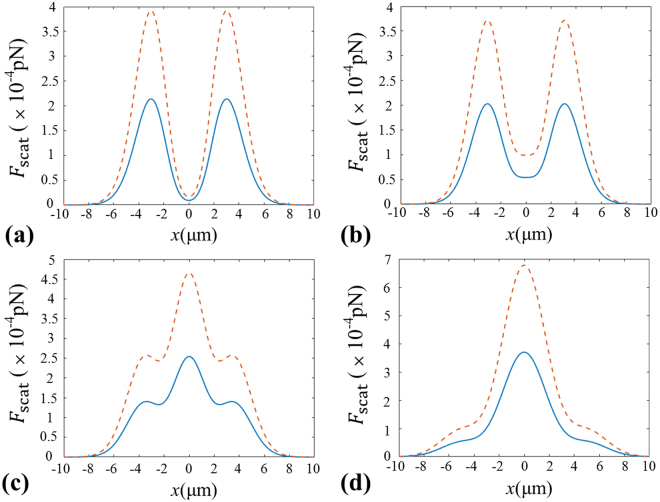
Scattering force produced by the focused beams generated by GMR at different planes. (**a**) *z* = 0 µm, (**b**) *z* = 5 µm, (**c**) *z* = 15 µm, (**d**) *z* = 30 µm. Solid curves for the particles with *n*_p_ = 1.59, dashed curves for the particles with *n*_p_ = 1.

**Figure 7:**
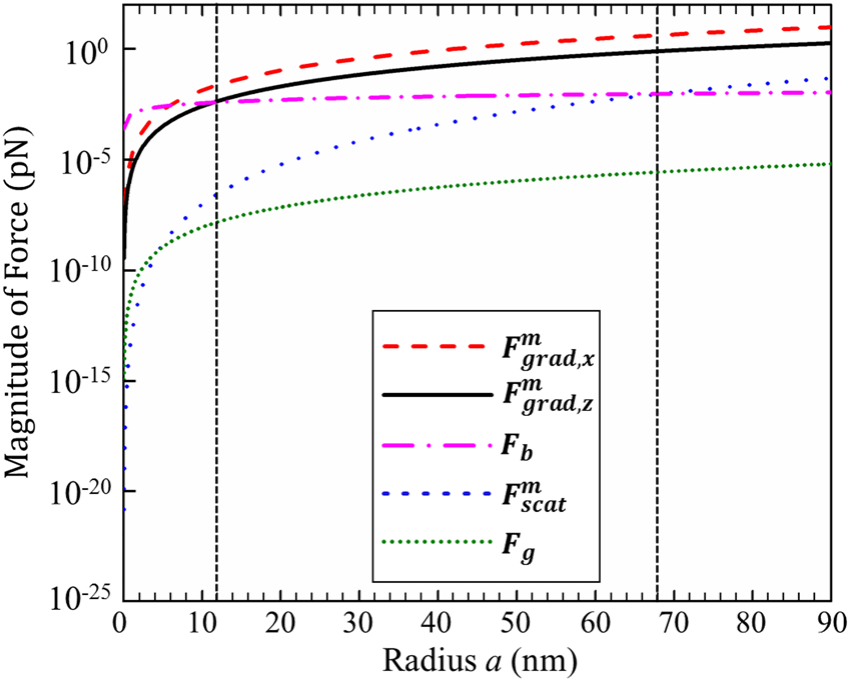
Magnitude of ${F}_{grad,x}^{{\rm{m}}}$(dashed red curve), ${F}_{grad,z}^{{\rm{m}}}$(solid black curve), *F*_*b*_ (dash-dotted pink curve), ${F}_{scat}^{{\rm{m}}}$(dotted blue curve) and *F*_*g*_ (dotted green curve) with different particles’ radii *a*.

From the above discussion, one can find that the radiation forces of the focused beams generated by GMR may be used to trap and manipulate the Rayleigh dielectric spheres. Under the Rayleigh approximation, it is well known that some conditions are required for stably manipulating the particles. Firstly, the longitudinal (axial) gradient force must be larger than the scattering force, i.e. $R=|{F}_{{\rm{grad}},z}|/|{F}_{{\rm{scat}}}|\ge 1$, where the ratio *R* is defined as the stability criterion. Figure 6 shows the scattering force at different propagation distances from the focus point. Compared with Fig. 4, one can easily find in Fig. 6 that the magnitude of scattering force is much smaller than the longitudinal gradient force. Secondly, the gradient force must overcome the effect of Brownian motion. For the convenience of comparison, Fig. 7 plots the magnitude of different forces versus particle’s radius *a*, in which ${F}_{grad,x}^{{\rm{m}}}$ represents the maximum transverse gradient force, ${F}_{grad,z}^{{\rm{m}}}$ denotes the maximum longitudinal gradient force, *F*_*b*_ is the Brownian force, ${F}_{scat}^{{\rm{m}}}$ is the maximum scattering force, and *F*_g_ is the gravity force. According to the fluctuation and dissipation theorem^34^, the Brownian force is defined as $|{F}_{b}|=\sqrt{12{\rm{\pi }}\eta a{k}_{B}T}$, here the viscosity of the water is *η* = 7.977 × 10^−4^ Pa∙s, *a* is the radius of particle, *k*_*B*_ is the Boltzmann constant, and the temperature *T* takes the value of 300 K. Compared with the gradient forces as shown in Fig. 7, the Brownian force is obviously far less than the radiation force. And it is found from Fig. 7 that the gravity of the particle could be neglected comparing with the gradient forces given the density of particles is 2 × 10^3^ kg/m^3^. Furthermore, one can see that for the case a < 12 nm the disturbance comes mainly from the Brownian motion, while the scattering force Fscat mostly affects trapping particles with a > 68 nm.

Another factor due to the Brownian motion will also strongly affect the trapping stability when the particles are very small. That is, the smaller the particle, the more difficult for stable trapping. In order to capture particles stably by radiation forces, the potential well induced by the gradient forces must be deep enough to overcome the kinetic energy of the particles. This condition can be given by using Boltzmann factor^1^: ${R}_{thermal}=\exp (-{U}_{\max }/{k}_{B}T)\ll 1$, where ${U}_{\max }={\rm{\pi }}{n}_{m}^{2}{\varepsilon }_{0}{a}^{3}|({\chi }^{2}-1)/({\chi }^{2}+2)|\cdot {|\mathop{E}\limits^{\rightharpoonup }|}^{2}$ represents the maximum depth of the potential well, *k*_*B*_ is the Boltzmann constant and *T* is the absolute temperature of the ambient. For the particles with χ > 1, the value of ${R}_{thermal}$ at the maximum intensity position is about *R*_thermal_ ≈ 0.02; For the particles with χ < 1, the value of ${R}_{thermal}$ at the maximum intensity position is about ${R}_{thermal}\,$ ≈ 0.0063. Obviously, all the values of Boltzmann factors near the focus are extremely small. Therefore the Brownian motion can be overcome or ignored in our case. In summary, we can conclude that the particles with 12 < *a* < 68 nm can be effectively confined and trapped by the focused GMR beams.

## Conclusions

In conclusion, we have investigated numerically and theoretically the radiation forces acting on a Rayleigh dielectric sphere produced by the beams generated by Gaussian mirror resonator (GMR). The results show that the focused beams generated by GMR can be used to trap and manipulate the particles with both high and low index of refractive nearby the focus point of the lens system. Also, the influences of optical parameters (e.g. *K* and *β*) of the beams generated by GMR on the radiation forces are discussed in detail. Finally, the conditions for capturing and manipulating the particle are analyzed under the Rayleigh approximation. Our results may have potential applications in biotechnology and nanoscience.
